# On the Use of the Electrospinning Coating Technique to Produce Antimicrobial Polyhydroxyalkanoate Materials Containing In Situ-Stabilized Silver Nanoparticles

**DOI:** 10.3390/nano7010004

**Published:** 2016-12-29

**Authors:** Jinneth Lorena Castro-Mayorga, Maria Jose Fabra, Luis Cabedo, Jose Maria Lagaron

**Affiliations:** 1 Novel Materials and Nanotechnology Group, IATA-CSIC, 46980 Valencia, Spain; jincasma@iata.csic.es (J.L.C.-M.); mjfabra@iata.csic.es (M.J.F.); 2 Polymers and Advanced Materials Group (PIMA), Universitat Jaume I, 12071 Castellón, Spain; lcabedo@uji.es

**Keywords:** electro-hydrodynamic processing, foodborne pathogens, metal nanoparticles, biopolyesters

## Abstract

Electro-hydrodynamic processing, comprising electrospraying and electrospinning techniques, has emerged as a versatile technology to produce nanostructured fiber-based and particle-based materials. In this work, an antimicrobial active multilayer system comprising a commercial polyhydroxyalkanoate substrate (PHA) and an electrospun PHA coating containing in situ-stabilized silver nanoparticles (AgNPs) was successfully developed and characterized in terms of morphology, thermal, mechanical, and barrier properties. The obtained materials reduced the bacterial population of *Salmonella enterica* below the detection limits at very low silver loading of 0.002 ± 0.0005 wt %. As a result, this study provides an innovative route to generate fully renewable and biodegradable materials that could prevent microbial outbreaks in food packages and food contact surfaces.

## 1. Introduction

Electro-hydrodynamic processing comprising electrospinning and electrospraying techniques is a broadly used and efficient technology that uses electrical forces to produce ultrathin fibers and nanocapsules, respectively [1,2]. Electrospinning has recently gained much attention not only because of its versatility in processing a wide range of polymer and biopolymer materials, but also because of its ability to produce fiber diameters within the submicro and nano range that is otherwise not viable to achieve by using conventional-generating technologies. The intrinsic characteristics of electrospun fibers, like very high specific surface and porosity [3] and the suitability of the technique to encapsulate active substances within the fibers, have prompted their use in a wide range of applications [4,5,6,7,8]. With the expansion of this technology, several researchers have used electrospinning to improve physicochemical and functional properties of biopolymer based materials by means of a controlled release of active compounds or by enhancing the dispersion of nano-additives into the biopolymer matrices [9,10,11].

Of particular interest is the generation of fiber-based systems for antimicrobial food packaging and food contact surface applications given that the direct application of biocide compounds onto the food surface can be inefficient because of their rapid diffusion within the bulk of food [12]. In this way, the incorporation of antimicrobial fiber-base mats as coating of the packaging material, leading to antimicrobial films, could improve their activity in maintaining an optimal effect during the food storage period. Furthermore, the high surface to volume ratio of electrospun mats could allow the efficient and prolonged delivery of previously loaded antibiotics [10].

Different antimicrobial compounds such as silver, ricinoleic acid [13], and others have been used to develop active food packaging materials either incorporated into the polymer matrix or directly copolymerized with the electrospun fibers. Due to their strong antimicrobial properties and thermal stability, silver nanoparticles (AgNPs) have so far been one of the most researched nanomaterials. The most common methods for the preparation of antibacterial and antifungal polymeric films have been direct blending of the nanometal with the matrix [14,15,16,17] and solvent casting [18,19,20,21]. However, the main challenge today is the synthesis of stable nanoparticles in a polymer solution, as their antimicrobial effectiveness depends on their size distribution and agglomeration state [22]. In general, when antimicrobials are used in applications related to pharma or biomedicine, the cytotoxicity of the materials becomes very relevant. Thus, there is increasing interest in the biological impact of AgNPs on a large scale and the possible risks to the environment and health [23]. Recent studies suggest that free-standing nanometer-sized particles may enter cells of living organisms, potentially leading to various cell injuries [24]. In this regard, Chairuangkitti and colleagues showed that silver contents below 25 ppm did not affect either the cell viability or the reactive oxygen species (ROS) generation of human lung carcinoma (A549) cells [25]. Also, Składanowski and collaborators concluded that AgNPs can be used as an antibacterial agent, being that these harmless for eukaryotic cells [26]; Salama et al. reached similar conclusion [27]. In any case, it is very unlikely that silver nanoparticles will be released from polymeric films that do not dissolve or strongly plasticize in the contact media [28].

On the incorporation of AgNPs into ultrafine fibers based on polymers, there are only a small number of studies on electrospun polymer fibers containing silver, such as poly(ε-caprolactone) [29], ethylene vinyl alcohol [11], poly-(l-lactide) [30], polypropylene [15], or polyacrylonitrile [31]. Further, to date, only few studies have yet been published about the preparation of silver and PHA based electrospun fibers [32,33], none of them analyzing the antimicrobial activity against foodborne pathogens. In a recent study, Castro-Mayorga et al. [34], demonstrated that the effective stabilization of AgNPs in an unpurified poly(3-hydroxybutyrate-co-18 mol %-3-hydroxyvalerate) (PHBV18) avoided the use of surfactants or capping agents prior to or during melt compounding with poly(3-hydroxybutyrate-co-3 mol %-3-hydroxyvalerate) (PHBV3). In that case, PHBV3/PHBV18 films, containing 0.04 wt % of AgNPs, showed a strong and sustained antibacterial activity against two of the most common foodborne pathogens, *Salmonella enterica* and *Listeria monocytogenes*. However, despite this progress, and especially considering that the application of these materials in the restrictive food legislation frames could be severely limited because of the use of AgNPs, the main goal of the present paper is the development of new strategies based on the coating design in order to reduce the AgNPs loading while keeping the antimicrobial performance.

The present work reports on the development and characterization of an antimicrobial multilayer system of polyhydroxyalkanoates (PHA) matrix and nanostructured silver-based coating obtained by means of the electrospinning technique. In particular, the active multilayer was characterized in terms of thermal, mechanical, and barrier properties and their antibacterial effectiveness against *S. enterica* and *L. monocytogenes* was also evaluated.

## 2. Materials and Methods

### 2.1. Materials

In this work, two different PHA grades, with different valerate (HV) contents, were used. PHBV3 (3 mol % valerate content, ENMAT Y1000P) was purchased in a pellet form from Tianan Biopolymers (Ningbo, China) and PHBV18 (18 mol % valerate content) was produced in a fermentation multi-stage process with mixed microbial culture and cheese whey industrial by-product as feed stock as it has been previously described by Martínez-Abad et al. [35].

### 2.2. Synthesis of Silver Nanoparticles in PHBV18 Matrices

The AgNPs were synthetized according to the in situ method reported by Castro-Mayorga et al. [36]. Briefly, 0.08 wt % of unpurified PHBV18 was suspended in ultrapure MilliQ^©^ water (Millipore Corporation Co., Billerica, MA, USA) and the assembly was placed into an ice bath and stirred using magnetic stirring. A sodium borohydride (Panreac, Barcelona, Spain) aqueous solution was added first to the suspension to get 2 mM concentration and then silver nitrate (>98% purity; Sigma-Aldrich, Hamburg, Germany) aqueous solution (1 mM) was added dropwise to generate AgNPs. Finally, the obtained product was separated by centrifugation and dried overnight in a vacuum oven at 40 °C.

### 2.3. Preparation of the Multilayer Systems

Multilayer films of PHBV3 and electrospun fibers with and without AgNPs were prepared as follows. The sample code and composition are summarized in Table 1.

#### 2.3.1. Preparation of the PHBV3 Film

PHBV3 films with a thickness of ca. 80 µm were prepared by compression-molding using a hot plate hydraulic press (Carver 4122, Wabash, IN, USA) at 180 °C and 1.8 MPa for 5 min.

#### 2.3.2. Preparation of the Electrospun Fibers

For the PHBVs and PHBVs/AgNPs electrospun fiber preparation, polymer solutions containing a total solids content of 6 wt % was prepared as follows: First, the PHBV3 at 92 wt % was dissolved in 2,2,2-Trifluoroethanol (TFE, ≥99 wt %, Sigma Aldrich, Hamburg, Germany) under magnetic stirring for 4 h at 50 °C and cooled down at room temperature. Then, PHBV18 or stabilized PHBV18/AgNPs were incorporated at the remaining 8 wt % and stirred for 12 more hours. Thereafter, the solution was processed using a Fluidnatek^®^ LE-500 pilot plant electrospinning setup manufactured by Bioinicia S.L., Valencia, Spain. The tool was operated under a steady flow-rate and made use of a motorized high throughput multinozzle injector, scanning vertically towards a metallic grid used as a collector. The distance between the needle and the collector was 20 cm, and the experiments were carried out at room temperature (23 ± 2 °C) for 15 min. The voltages of the collector and injector were set at 24 kV and −18 kV, respectively. The flow rate was 80 mL/h. After electrospinning, the fiber mats were dried at 40 °C under vacuum overnight to completely remove the solvent and were subsequently used to prepare the coated systems.

#### 2.3.3. Preparation of the Multilayer Films

PHBV3 films were coated with PHBVs and PHBVs/AgNsP ultrathin fiber mats produced by means of electrospinning and an annealing step was applied. Fiber mats were placed onto PHBV3 films and the assembly was put in between hot plates hydraulic press (Carver 4122, Wabash, IN, USA) at 160 °C for 2 min (without pressing). The total amount of electrospun coating (~0.71 ± 0.01 mg/cm^2^) was estimated by weighing the coated system before and after deposition of the electrospun material which corresponded to ~5 wt % of the coated systems.

### 2.4. Determination of Silver Content in the Active Multilayer Systems

The total Ag content within the active multilayer systems was determined by subjecting 100 mg samples to acid digestion with 2 mL of Hiperpur HNO_3_ (Panreac, Barcelona, Spain) at 80 °C overnight. The resultant digestant was diluted to a final volume of 5 mL. The quantification of silver was carried out by inductively coupled plasma-optical emission spectroscopy (ICP-OES, Perkin-Elmer, Waltham, MA, USA) using a silver standard solution (traceable to SRM from NIST, AgNO_3_ in HNO_3_ 2%–3%, 1000 mg/L Ag Certipur^®^, Merck, Darmstadt, Germany) for calibration. All measurements were done, at least, in triplicate.

### 2.5. Transmission Electronic Microscopy (TEM)

The morphology of the electrospun fibers and the active multilayer films was studied using a Jeol 1010 (Hitachi, Tokyo, Japan), transmission electronic microscope an accelerating voltage of 80 kV. Samples were previously ultra-microtomed (Leica EM UC6, Wetzlar, Germany) and placed onto carbon-coated copper grids.

### 2.6. Scanning Electronic Microscopy (SEM)

SEM was conducted on a Hitachi S-4800 microscope (Hitachi, Chiyoda, Tokyo, Japan) at an accelerating voltage of 5 kV and a working distance of 8–10 mm. The active multilayer films were cryo-fractured after immersion in liquid nitrogen and subsequently sputtered with a gold-palladium mixture under vacuum before their examination. Estimation of the average fiber diameter was done by means of the Adobe Photoshop CS4 software from 300 fibers at random from SEM images.

### 2.7. Differential Scanning Calorimetry (DSC)

Thermal properties of the neat electrospun fibers, PHBV3 films and the multilayer systems were evaluated by Differential Scanning Calorimetry (DSC) using a Perkin-Elmer DSC 8000 (Waltham, MA, USA) thermal analysis system under nitrogen atmosphere. The analysis was carried out on ~3 mg of each sample at a heating rate of 10 °C/min, from 0 °C to 200 °C, followed by a subsequent cooling down to −50 °C. The DSC equipment was calibrated with indium as a standard and the slope of the thermograms was corrected by subtracting similar scans of an empty pan. Tests were done, at least, in triplicate.

### 2.8. Mechanical Properties

Tensile tests of the neat PHBV3 and the multilayer systems were carried out in a universal testing machine (Shimadzu AGS-X 500N, Kyoto, Japan) at a crosshead rate of 10 mm/min at room temperature. All samples were allowed to reach the equilibrium under ambient conditions (25 °C and 50% R.H. for 24 h before the testing). Tests were performed according to ASTM D638 with dumb-bell samples die-cut from prepared films. Elastic Modulus (E), Tensile Strength (TS), and Elongation at Break (EAB) were determined from the stress-strain curves, estimated from force–distance data obtained for the different films.

### 2.9. Barrier Properties

#### 2.9.1. Water Vapor Permeability (WVP)

WVP was measured, in triplicate, according to the ASTM E96 gravimetric method, using Payne permeability cups (Elcometer SPRL, Ourpeye, Belgium). Distilled water was placed inside the cup to expose the film (the exposed area was 9.6 × 10^−4^ m^2^) to 100% RH on one side. Once the films were secured, each cup was placed in an equilibrated relative humidity cabinet at 0% RH and room temperature. The cups were weighed periodically (±0.0001 g). Water vapor permeation rate was calculated from the steady-state permeation slopes (eight points) obtained from the regression analysis of weight loss data vs. time, and weight loss was calculated as the total cell loss minus the loss through the sealing. Permeability was obtained by multiplying the permeance by the average film thickness.

#### 2.9.2. Oxygen Permeability (PO_2_)

The PO_2_ was derived from oxygen transmission rate (OTR) measurements recorded using an Oxygen Permeation Analyzer M8001 (Systech Illinois, Thame, UK). Experiments were carried out at 23 °C and 80% RH. The samples were previously purged with nitrogen in the humidity equilibrated samples, before exposure to an oxygen flow of 10 mL/min. The exposure area during the test was 5 cm^2^ for each sample. In order to obtain the oxygen permeability, film thickness and gas partial pressure were considered in each case. The measurements were done in triplicate.

### 2.10. Antimicrobial Activity of Coated Systems

Gram negative, *Salmonella enterica* CECT 4300 and Gram positive *Listeria monocytogenes* CECT 7467 were used to determine the antimicrobial activity of the obtained films. The strains were purchased from the Spanish Type Culture Collection (CECT: Valencia, Spain) and stored in phosphate buffered saline (PBS, Sigma Aldrich, St. Louis, MO, USA) with 10 wt % tryptic soy broth (TSB, Conda Laboratories, Madrid, Spain) and 10 wt % glycerol at −80 °C until needed. For experimental use, a loopful of bacteria was transferred to 10 mL of TSB and incubated at 37 °C overnight and an aliquot was again transferred to TSB and grown at 37 °C and 120 rpm to the mid-exponential phase of growth having an absorbance value of 0.20 as determined by optical density at 600 nm (Agilent 8453 UV-visible spectrum system, Santa Clara, CA, USA). This culture served as inoculum for antimicrobial assays.

To perform the susceptibility study, a modification of the Japanese Industrial Standard JIS Z 2801 (ISO 22196) was used. Briefly, a microorganism suspension containing about 5 × 10^5^ CFU/mL was applied onto the active multilayers of 3 × 3 cm and covered by an inert piece of Low-Density Polyethylene (LDPE) of 2.5 × 2.5 cm and 80 µm of thickness. After incubation at room temperature and a relative humidity of at least 95% for 24 h, bacteria were recovered with PBS, and the viable cells determined by the conventional plate count method. The multilayer film (without AgNPs) was used as a negative control. Three specimens of each sample were tested.

### 2.11. Statistical Analysis

Statistical analysis of physicochemical and antimicrobial data was performed through analysis of variance (ANOVA) using StatGraphics Plus for Windows version 5.1 (Statistical Graphics Corporation, Princeton, NJ, USA). Homogeneous sample groups were obtained by using Tukey’s Honestly Significant Difference (HSD) (95% significant level). Data were reported as mean values ± standard deviation.

## 3. Results and Discussion

### 3.1. Morphology

It is well-known that efficient electrospinning depends on the solution properties (typically viscosity, surface tension, and conductivity). In this sense, stable electrospinning is often only achieved when the viscosity is high enough to produce the necessary polymer entanglements to form fibers and one of the most important factors which govern this aspect is the polymer concentration in the electrospinning solution. Based on screening studies dealing with the electrospinning of PHBVs, the concentration of the total polymer was adjusted to obtain stable electrospinning process avoiding the dripping of the solutions, the formation of beaded areas, or the formation of fibers in which the average diameters surpassed the micron size. Therefore, taking into account all these aspects, the concentration of the PHBVs was set to 6 wt %, as to obtain continuous fibers with no beads and in the submicron diameter range. For comparative purposes, the PHBV3/PHBV18 ratio was established at 92:8, according to the mixture used for highly efficient antibacterial nanocomposites recently reported by Castro-Mayorga et al. [36].

**Figure 1:**
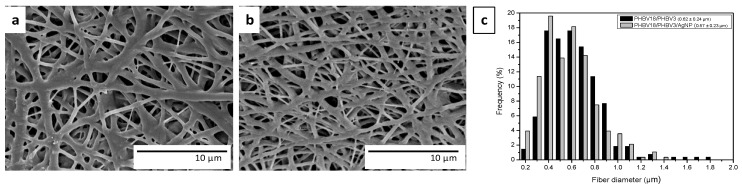
Scanning electron microscopy (SEM) images of the electrospun fibers: (**a**) PHBVs (PHBVs corresponds to a mixture between PHBV3 and PHBV18 (without silver nanoparticles), PHBV3, poly(3-hydroxybutyrate-co-3 mol %-3-hydroxyvalerate), PHBV18, poly(3-hydroxybutyrate-co-18 mol %-3-hydroxyvalerate)); (**b**) PHBVs/ silver nanoparticles (AgNPs); (**c**) size distribution of fibers.

SEM micrographs of the PHBVs fibers with and without AgNPs and their corresponding average diameters are shown in Figure 1. The first clear observation is that the addition of AgNPs did not significantly modify the morphology of the fibers probably due to their low concentration in the PHBV mixture. In contrast, several authors have reported that the addition of silver ions or AgNPs greatly affected the diameter of the fibers, causing a significant reduction in the average diameter. However, in those cases, the additive was added in higher concentrations. They reported that the presence of silver, either in the form of ions or nanoparticles, increased the charge density and conductivity, which produces an increase in the stretching forces in the jet, consequently decreasing the fiber diameter [11,37,38].

**Figure 2:**
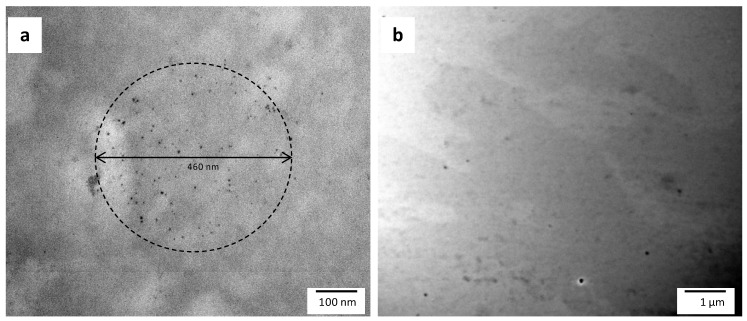
Transmission electron microscopy (TEM) micrographs of ultramicrotomed silver-containing materials: (**a**) in PHBVs/AgNPs; (**b**) in the active multilayer film. The dashed line in the Figure 2a shows a group of AgNPs whose diameter matches with the fiber’s diameter of 0.46 µm.

In order to obtain more detailed information about the dispersion and distribution of individual AgNPs into the electrospun fibers and into the active multilayer system, these samples were ultra-microtomed and analyzed by TEM. Figure 2 shows the TEM micrographs of an ultrathin section of electrospun PHBVs/AgNPs fibers (Figure 2a) and microtomed thin sheets of the active multilayer (Figure 2b). It was clearly observed that AgNPs were well dispersed into the electrospun fibers and into the active multilayer, having an average particle diameter of 6 ± 1 nm. Since, by TEM observation, only the nanoparticles can be seen and not the fibers, it is interesting to note that the diameter enclosing the AgNPs observed in Figure 2a matches the actual electrospun fiber’s diameter (ca. 0.46 µm), hence suggesting the AgNPs are well dispersed and distributed in the electrospun fibers. Thus, the PHBV18 clearly favored a proper entrapment of highly dispersed and distributed AgNPs. Compared with other PHBVs/AgNPs nanocomposites, the electrospinning process results in a lack of agglomeration that more easily occurs when processed by conventional melt compounding methods [15,36,39].

**Figure 3:**
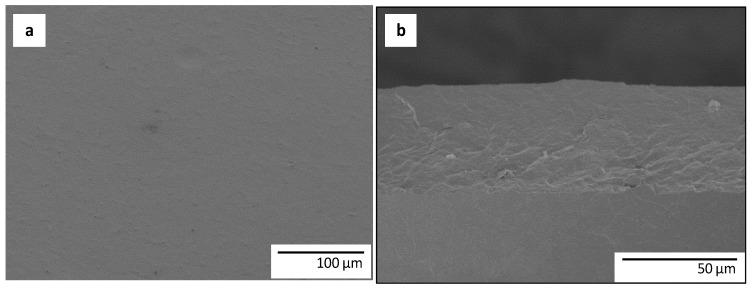
SEM micrographs of the active multilayer; (**a**) top view of the film; (**b**) side view of the cryo-fractured film section.

The morphology of the multilayer structures based on PHBV3 and PHBVs/AgNPs electrospun coatings were also examined by SEM and representative images are displayed in Figure 3. As it can be seen from this figure, after the annealing step, the active multilayer films exhibited a continuous and smooth surface from a top view ( Figure 3a), which is strongly adhered to the bottom layer in the cryo-fractured side view ( Figure 3b), revealing the excellent adhesion between the two layers after the annealing step.

**Figure 4:**
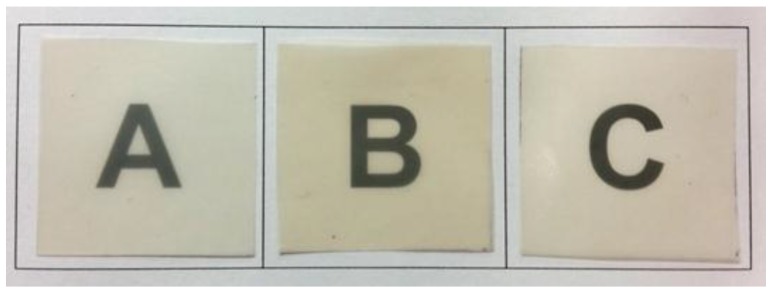
Contact transparency pictures of films. (**A**) poly(3-hydroxybutyrate-co-3 mol %-3-hydroxyvalerate) (PHBV3); (**B**) multilayer; (**C**) active multilayer.

Figure 4 shows the overall contact transparency of the neat PHBV3 (Figure 4a) and of the multilayers prepared without and with AgNPs (Figure 4b,c, respectively). As observed, the coated systems preserved a good contact transparency and neither the multilayer nor the active multilayer induced significant differences in appearance in the internal transmittance of the films (data not shown). Therefore, it could be stated that no significant degradation of PHBV18 or silver reduction/agglomeration events occurred during the annealing step.

### 3.2. Thermal Properties

**Figure 5:**
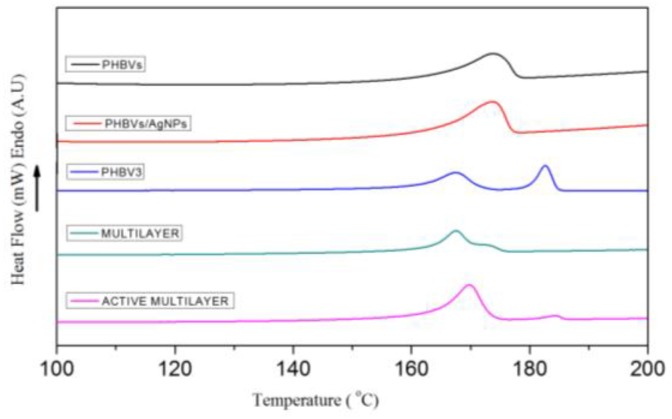
Differential scanning calorimetry (DSC) thermograms of first heating run of the neat electrospun fibers and films and their silver-based coating system.

With the aim of investigating the effects of AgNPs addition and PHBVs/AgNPs electrospun coating on the thermal properties, differential scanning calorimetry (DSC) analyses of neat and silver-base fibers as well as PHBV3 layer and bilayer systems with and without AgNPs were carried out (Table 2 and Figure 5). The melting point (*T*_m_) and melting enthalpies (Δ*H*_m_) were calculated from the maximum temperatures and peak area, respectively, of the peak associated with the melting process from the first heating runs. The crystallization temperature (*T*_c_) was also obtained from the cooling run. From Table 2, the presence of the silver nanoparticles seems to modify only slightly up the *T*_c_, perhaps suggesting a small nucleation effect also reflected in the slightly increased melting enthalpy; albeit the changes are not statistically significant.

However, from observation of Figure 5, it is clear that the formulation of the materials does have an effect in the shape of the melting endotherms of the different samples that is clearly related to the fact that electrospun fibers and electrospun coated films do have a different melting behavior during the DSC run. These variations can be ascribed to the morphology of the fibers which, during melting, seem to behave more homogeneously due to the high surface to volume ratio of the electrospun materials as compared to a thicker continuous film. The coated samples also show a different behavior as compared to the bulk PHBV3 compression molded film which suggests that the lower melting point of the coating alters the overall melting behavior of the bilayer. Thus, the observed multiple melting endotherms are here found to depend on how the samples were obtained and not on the actual intrinsic polymer composition. Therefore, the results above support that melting recrystallization phenomena during the DSC run could be at the origin of the multiple endotherms for this polymer as previously suggested in the literature [35,36,40,41].

### 3.3. Barrier Properties

Table 3 gathers water vapor and oxygen permeability of the PHBV3 matrix and the coated systems with or without AgNPs. In general, the presence of the PHBVs coatings did not significantly alter the barrier properties of the neat PHBV3 film. Although the neat PHBV18 film has been reported to have higher oxygen and water vapor permeability values than the neat PHBV3 [36], its incorporation as a part of the electrospun coating did not significantly affect barrier properties of the PHBV3, probably due to the low concentration used. Contrary to that observed for nanocomposites prepared with PHBV3/PHBV18/AgNPs [36], the addition of the stabilized AgNPs did not result in a positive barrier effect on the active multilayer. These differences could be explained by the low AgNPs loading which was not enough to create a meaningful blocking tortuous path in the PHBV matrix. In contrast, Kanmani and Rhim [42] reported that the reduction in water vapor (WVP) of the gelatin-based nanocomposite films containing AgNPs was mainly due to nanofillers causing a more tortuous pathway for water vapor diffusion. This indicates that the barrier efficiency of nanocomposite films containing AgNPs, depends on having a sufficient quantity of nanoparticles since the spherical shape of this nanofiller is not thought to be as efficient in reducing diffusion as plate-like fillers such as clay minerals.

### 3.4. Mechanical Properties

The materials’ Elastic Modulus (E), Elongation at Break (EAB) and Maximum Tensile Strength (TS) are presented in Table 4. The neat PHBV3 film presented an excellent rigidity and strength but an excessive brittleness. The mechanical performance of PHBV3 was not altered by the incorporation of electrospun PHBVs coating with or without AgNPs. The active multilayer seems to be somewhat more ductile than its counterparts prepared without AgNPs, although differences were not found significant. The lack of effect on mechanical properties in the coated systems was in line with the previous observations by Castro-Mayorga et al. [35] and Jeong et al. [15] in which highly dispersed AgNPs did not present a significant effect on PHBV melt compounded nanocomposites. However, once again, the AgNPs loading was perhaps too low to impact the mechanical performance of the neat polymer.

### 3.5. Antimicrobial Activity

**Figure 6:**
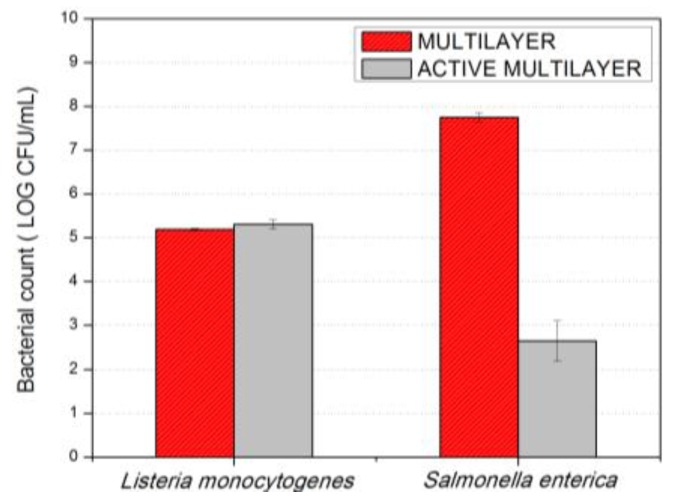
Antimicrobial activity of PHBV and PHBV/AgNP films against *Listeria monocytogenes* and *Salmonella enterica* after 24 h of exposure. The initial inoculum size was ~5 log CFU/mL and the detection limit was 20 CFU/mL.

To evaluate the antimicrobial activity of the coated systems containing AgNPs on the surface, a modification of the Japanese Industrial Standard JIS Z 2801:2000 (ISO 22196) was followed, the results are presented in Figure 6. The incubation temperature of 23 ± 2 °C was chosen to mimic room temperature conditions of possible application of these plastics in food contact or other antimicrobial surfaces. The first observation to highlight is that, after 24 h of exposure, the active multilayer did not show any antibacterial effect against *Listeria monocytogenes*. In contrast, a surprising reduction of ca. 5 log CFU/mL of *Salmonella enterica* was recorded. These differences are in line with previous studies reporting a higher susceptibility of gram-negative as compared with gram-positive bacteria [43,44] which related this finding with the presence of multiple layers of peptidoglycan-containing teichoic acids or lipoteichoic acids with strong negative charge that may contribute to sequestration of free Ag ions needed to exert the antimicrobial effect.

It should also be noted that, on the silver-based antimicrobial materials, Castro-Mayorga et al. [35] have recently demonstrated the bactericide effect of melt compounded nanocomposites containing 0.040 ± 0.002 wt %, AgNPs against both *Listeria monocytogenes* and *Salmonella enterica*. However, in the present work, the total silver content was 20 times lower than the previously cited report (i.e., of only 0.002 ± 0.0005 wt % as measured by ICP-OES) and even more than 50 times lower than other ones reporting silver loading between 0.1 and 5 wt % [14,19,32,45]. This fact represents not only a significant reduction of silver concentration required to achieve a bactericide effect against *Salmonella enterica* but also an alternative way to satisfy the specific limit of migration established by the current legislation.

A similar set of samples used for the antimicrobial tests described above are currently being tested with regard to their cytotoxicity; the preliminary results (not shown) suggest that the materials do not provide cytotoxic effect when tested in direct contact with colorectal carcinoma cells (HCT116) or with neonatal non-tumor fibroblasts cells (the study when completed will be published elsewhere).

## 4. Conclusions

The use of the electrospinning coating technique allows the development of packaging materials with unique properties. The present work successfully reports on the potential of the annealed electrospun coating process based on AgNPs and PHA as efficient antimicrobial materials, showing that the antimicrobial performance can be enhanced compared to the direct melt-compounded process previously reported. In fact, by means of the active electrospun coating, lower silver loadings are needed to achieve a bactericidal effect against *Salmonella enterica*. Furthermore, the physicochemical properties of the neat PHBV3 polymer were not detrimentally affected by the presence of the silver-based coating. Thus, the results of this work provide a new route to generate more efficient antimicrobial heterogeneous-across-thickness films that can be of relevance to develop active multilayer materials for food packaging and food contact surface applications.

## Figures and Tables

**Table 1:** Sample code and composition of the developed materials.

Sample code	Composition
poly(3-hydroxybutyrate-co-3 mol %-3-hydroxyvalerate) (PHBV3)	100% commercial PHBV3
PHBVs (a mixture of PHBV3 and PHBV18)	92% commercial PHBV3 + 8% Mixed microbial culture derived poly(3-hydroxybutyrate-co-18 mol %-3-hydroxyvalerate) (PHBV18)
PHBVs/silver nanoparticles (AgNPs)	PHBVs + Silver nanoparticles
Multilayer	Substrate PHBV3 + PHBVs coating
Active Multilayer	Substrate PHBV3 + PHBVs/AgNPs coating

**Table 2:** Differential scanning calorimetry (DSC) parameters of the neat electrospun fibers and films and their silver-based coating system.

Sample	*T*_m1_ (°C)	*T*_m2_ (°C)	*T*_c_ (°C)	Δ*H*_m_ (J/g)
PHBVs	173.7 ± 0.2 ^a^	-	113.4 ± 0.5 ^a^	61 ± 1 ^a^
PHBVs/AgNPs	173.8 ± 0.1 ^a^	-	114.9 ± 0.3 ^a^	65 ± 1 ^a^
PHBV3	168.7 ± 1.0 ^b^	181.8 ± 0.2 ^a^	114.7 ± 0.0 ^a^	72 ± 1 ^b^
Multilayer	168.5 ± 1.4 ^b^	-	117.3 ± 0.1 ^b^	73 ± 1 ^b^
Active Multilayer	170.4 ± 0.8 ^a,b^	184.8 ± 0.7 ^b^	117.4 ± 0.6 ^b^	74 ± 1 ^b^

Mean values ± standard deviation. Mean values with different superscript letters in the same column represent significant differences (*p* < 0.05) among the samples according to ANOVA and Tukey’s multiple comparison tests.

**Table 3:** Water vapor (WVP) and oxygen permeability (PO_2_) measurements of the neat PHBV3 and PHBVs films and the silver-based coating systems.

Sample	WVP (Kg·m/Pa·s·m^2^)	PO_2_ (m^3^·m/m^2^·s·Pa) 80% RH
PHBV3	(1.10 ± 0.02) × 10^−15^ ^a^	(2.06 ± 0.09) × 10^−19^ ^a^
Multilayer	(1.25 ± 0.25) × 10^−15^ ^a^	(2.13 ± 0.12) × 10^−19^ ^a^
Active Multilayer	(1.59 ± 0.38) × 10^−15^ ^a^	(2.17 ± 0.19) × 10^−19^ ^a^

Mean values ± standard deviation. Mean values with different superscript letters in the same column represent significant differences (*p* < 0.05) among the samples according to ANOVA and Tukey’s multiple comparison tests.

**Table 4:** Tensile parameters of the neat PHBV3 and PHBV films and the silver-based coating systems.

Sample	E (GPa)	EAB (%)	TS (MPa)
PHBV3	2.6 ± 0.1 ^a^	1.5 ± 0.2 ^a^	33.9 ± 6.9 ^a^
Multilayer	2.6 ± 0.2 ^a^	1.8 ± 0.2 ^a^	37.1 ± 2.8 ^a^
Active Multilayer	2.6 ± 0.1 ^a^	2.4 ± 0.6 ^a^	29.9 ± 3.5 ^a^

Mean values ± standard deviation. Mean values with different superscript letters in the same column represent significant differences (*p* < 0.05) among the samples according to ANOVA and Tukey’s multiple comparison tests.
